# Co‐Designing a Peer Navigator Role to Improve Equity in Healthcare Access for Pacific Islander, Māori and Arabic Communities in Australia

**DOI:** 10.1111/hex.70351

**Published:** 2026-01-29

**Authors:** Lubab Shwaita, Simone Said, Susan D. Whicker, Fan Hoak, Lizcha Kaivaha, Wadeed Salboud, Charan Stevenson, Mokhaled Mohammed, Yue Hu, Rebecca L. Jessup

**Affiliations:** ^1^ Staying Well and Hospital Without Walls Program, Northern Health Melbourne Australia; ^2^ Transcultural and Language Services, Northern Health Melbourne Australia; ^3^ Victorian Centre for Virtual Health Research, Northern Health Melbourne Australia; ^4^ School of Allied Health, Human Services and Sport La Trobe University Melbourne Australia

**Keywords:** access, co‐design, communication, culturally and linguistically diverse, language barrier, navigation, peer health navigation

## Abstract

**Background:**

There is a great deal of variation in the design and delivery of the peer health navigator role, making it difficult to adapt role responsibilities into context. In this study, we aimed to co‐design a bicultural peer health navigator (BPHN) role to meet the needs of culturally and linguistically diverse (CALD) people from Pacific Islander, Māori and Arabic‐speaking backgrounds.

**Methods:**

A two‐phase co‐design approach involving workshops with follow‐up member checking via interview was used to gain insight into factors affecting patient interaction and access barriers to health services.

**Results:**

Barriers described by participants emerged under five major themes—overcoming language barriers, improving communication, navigation and access to information, appointment reminders, and health and social services education in the community.

**Conclusion:**

Clear tasks for the BPHN role were identified by the participants to improve accessibility and usage of healthcare services. Future work should involve feasibility testing with the support and involvement of community members, BPHNs and their supervisors and importantly health service leaders.

**Patient or Public Contribution:**

Four of the authors were employed as bicultural peer health navigators (BPHNs) who engaged their communities to organise and conduct these workshops in their preferred language. This approach enabled an inclusive environment for the participants to share their thoughts and experiences. The proposed roles for a BPHN were generated from the workshop discussions. The BPHNs conducted the semi‐structured verification interviews with the workshop participants. The thematic analysis was conducted by two of the BPHNs, with one being the primary author.

## 1 Background

Australia is one of the most culturally and linguistically diverse (CALD) countries in the world [1]. The term CALD, utilised by the Australian Bureau of Statistics, refers to people born overseas, those with a parent born overseas or those who speak a language other than English at home [2]. CALD communities represent 27.6% of the Australian population, with a further 48.2% having a parent born overseas and 22% speaking a language other than English at home [3].

Although Australia is home to a substantial and growing CALD population, disparities in health outcomes persist, highlighting the complex relationship that exists between cultural diversity and systemic health inequities. CALD communities experience higher rates of preventable hospitalisations, lower cancer screening participation [4] and delayed access to essential services compared to non‐CALD populations [5]. These inequities are not uniform across all CALD groups and often intersect with visa status, socio‐economic disadvantage, negative provider experiences and systemic racism [6, 7, 8]. Contributing to these challenges is the inherent complexity and fragmentation of the system itself, which makes it additionally difficult for CALD communities to understand how to access and navigate to the right care at the right time [5]. These challenges are exacerbated by lower health literacy, language and communication barriers, and limited awareness of available services. These inequities can lead to poorer engagement, poorer experiences of care and poorer health outcomes in CALD communities [9, 10, 11].

One strategy to help overcome health access barriers is the establishment of bicultural peer health navigator (BPHN) roles [12]. Several Australian studies have explored the role of health navigation in addressing these disparities and have shown promise in enhancing service engagement and health literacy amongst migrant and refugee communities [13, 14, 15]. The concept of health navigation is not new and originated in the United States in 1990 to support African American women in accessing free breast cancer screening, examination and earlier diagnosis [16]. While originally conceptualised as a professional ‘nurse navigator’ role, over time the roles have developed to include ‘lived experience’ peer health navigators working in condition‐specific areas such as cancer and mental health and for specific services such as emergency departments and primary healthcare [17]. Unlike clinical staff, peer health navigators do not provide healthcare services directly, but instead offer person‐centred health and social care navigation by aiding and guiding individuals to help overcome barriers to care. BPHN roles add an important cultural lens, building trust through shared language, culture and lived experience [9, 18, 19, 20, 21].

Internationally, peer health navigators have been shown to improve healthcare access and engagement among underserved populations [22, 23]. However, their roles and responsibilities are typically context‐specific, and their success often hinges on cultural alignment between the navigator and the individuals they assist. In 2022, the World Health Organization released a policy brief that highlighted examples where BPHNs contributed not only to improved healthcare access but also to enhanced prevention and health promotion efforts within hard‐to‐reach communities [17]. The brief emphasised that while patient navigation programmes can yield positive outcomes, their effectiveness depends on local adaptation, and roles are not directly transferable across settings or countries. For health services in Australia, this presents an opportunity by learning from international success, while tailoring roles to local needs, BPHNs can be positioned to bridge cultural divides and improve healthcare experiences and outcomes for diverse populations.

During the Covid‐19 pandemic, Pacific Islander, Māori and Arabic‐speaking communities experienced disproportionately poorer health outcomes than other CALD populations and Australian‐born individuals [24]. Specifically, when compared to people who were born in Australia, Pacific Islander‐born communities experienced a mortality rate up to 80 times higher, while Arabic‐speaking communities experienced a mortality rate 13 times higher [25]. Public health data and frontline experiences revealed lower vaccination rates, delayed care‐seeking when unwell, and limited engagement with existing health services in these populations [26]. These patterns were not due to individual behaviours alone but reflected deeper systemic issues, including limited reach of public health messaging, gaps in culturally tailored care and a lack of trust or connection with health institutions [24].

As Australia's population continues to diversify, and as the healthcare system grows more complex, addressing long‐standing barriers to access becomes increasingly urgent [3, 27]. Although peer health navigators have demonstrated success internationally, evidence of their effectiveness in the Australian context remains limited, and their implementation is inconsistent. There remains a need for culturally specific, co‐designed health navigator roles that consider the unique structural and cultural barriers faced by diverse CALD subgroups within the Australian healthcare system. In response to the challenges faced at our health service, combined with the need for greater exploration of what a BPHN role might look like in the Australian hospital setting, we sought to work alongside community members and employed BPHNs to identify barriers to access and to collaboratively design solutions grounded in cultural strengths, lived experience and local priorities. The aim of this study was therefore to identify the barriers to healthcare access faced by Pacific Islander, Māori and Arabic‐speaking communities living in the north of Melbourne and to co‐design practical solutions that could be delivered by BPHNs.

## 2 Materials and Methods

### 2.1 Study Design

A co‐design approach to identify gaps and barriers to healthcare access and propose responsibilities for Pacific Islander, Māori and Arabic‐speaking BPHNs to address access issues in health service settings.

### 2.2 Ethical Considerations

Research ethics and institutional governance were sorted, and approval was provided. The guidelines of the Declaration of Helsinki were followed in every stage of this study. All participants provided their informed consent to participate in this study.

### 2.3 Participants and Setting

We recruited participants from Pacific Islander, Māori and Arabic‐speaking communities residing in the northern suburbs of Melbourne who had either attended or were present when a family member attended Northern Health for healthcare. Arabic‐speaking participants originated from Lebanon, Iraq and Syria. Northern Health is a major provider of hospital and ancillary‐related healthcare services in the north of Melbourne. Northern Health is the primary provider of healthcare in Melbourne's northern region and covers an area of high diversity, with over 185 languages and 128 countries of origin identified [28].

### 2.4 Procedures and Data Collection

We employed purposive sampling to recruit participants through community organisations which offer support across a wide variety of contexts, including migrant support, family services, counselling, advocacy and community programmes.

To recruit participants, the BPHNs met with the leaders of the community organisations to discuss the relevance of the study to their membership. A summary of the study was also provided to support participant recruitment. Two Arabic‐speaking community organisations were approached to assist in the recruitment of participants. Pacific Islander and Māori participants were recruited through community sports clubs and church congregations. Participants needed to be over 18 years old, identify as members of an Arabic‐speaking community or be of Pacific Islander or Māori origin, reside in the Northern Health catchment area and be able to provide informed consent. We sought diversity in age, gender, educational level and the number of years residing in Australia. Participants were excluded if they were under 18 years of age. Participants were provided with the Participant Information and Consent Form for review before the day of the workshop and provided verbal consent on the day. All workshops were facilitated by the BPHNs, and a light meal was provided. The Arabic‐speaking participant workshops were conducted in Arabic to ensure participants could engage fully and comfortably in discussions, given that many community members had limited English proficiency. These were supported by a National Accreditation Authority for Translators and Interpreters (NAATI) [29] certified Arabic‐speaking interpreter. The Pacific Islander and Māori workshops, which included participants from Tongan, Samoan, Niuean, Cook Islands and New Zealand Māori backgrounds, were conducted in English. This was due to English being the shared language among the group, with all participants demonstrating high proficiency. Holding the sessions in the most accessible and inclusive language for each group supported meaningful engagement and culturally safe dialogue across all workshops.

To guide discussion while allowing flexibility, we used seeding questions and scenarios with open‐ended prompts designed to initiate dialogue around key topics without leading participant responses. These were developed in collaboration with bicultural health workers to ensure cultural relevance and sensitivity. This approach helped anchor discussions in lived experience and encouraged participants to reflect on barriers and potential solutions in a way that was meaningful to them. Box 1 provides an example of a scenario developed to facilitate discussion in each of the co‐design workshops.

Box 1 **Scenario example used to facilitate discussion in the co‐design workshops.**

Imagine you are 70 years old. You have several health problems, including diabetes and high blood pressure. Recently, you received a letter in the mail about a medical appointment at the hospital. However, you're unsure why you have received this appointment. Is this a situation that you have ever found yourself in? Or has this happened to someone that you know? What would make you feel more confident about accessing the healthcare system? What other barriers might they face? What things might a person need to help them overcome those barriers? Would a solution look similar or different if it were addressing the needs of a young pregnant mother trying to decide how to best access healthcare services during her pregnancy?

Following the workshop, participants were invited to participate in a follow‐up interview by telephone, where themes and suggested responsibilities for the BPHN role arising from the workshops were presented. This process was used as a form of member checking. By inviting participants to confirm or clarify the themes that emerged from the workshops, we aimed to not only ensure the trustworthiness of our analysis but also demonstrate our respect for participant voice and agency. Participants were offered a gift card for attending a workshop and an additional gift card for completing the follow‐up telephone interview. All participants from the workshops participated in a follow‐up interview with a BPHN.

**Figure 1:**
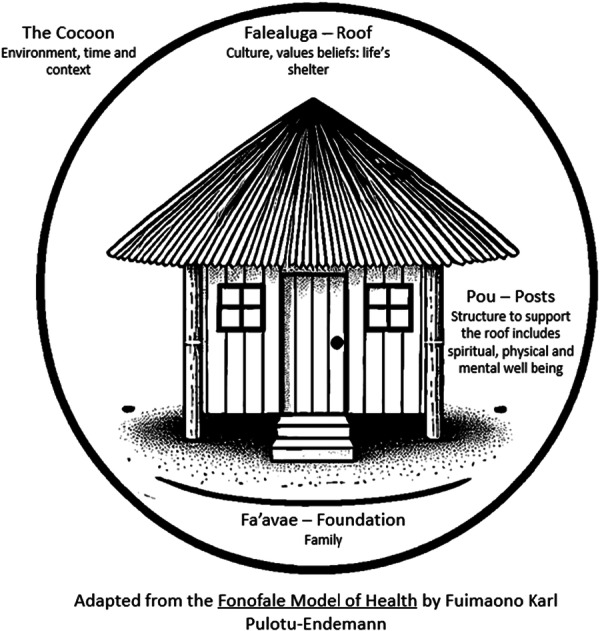
Fonofale model used to set the scene for Pacific Islander and Māori workshops.

Overall, six workshops with a total of 46 participants were conducted (Table 1). Each workshop lasted around 60 min. Four workshops were conducted with members from two Arabic community organisations in their meeting rooms. Two workshops were conducted with members from Pacific Islander and Māori communities in a community hub. For the workshops with Pacific Islander and Māori participants, issues raised were addressed within the context of the Fonofale model by Fuimaono Karl Pulotu‐Endemann in 1984 [30, 31]. The Fonofale model is a cultural framework for the health and well‐being of individuals of Pacific Island cultural background (Figure 1).

**Table 1:** Demographics of workshop participants.

	Workshop 1	Workshop 2	Workshop 3	Workshop 4	Workshop 5	Workshop 6
	Arabic‐speaking	Arabic‐speaking	Arabic‐speaking	Arabic‐speaking	New Zealand Māori and Pacific Islander	New Zealand Māori and Pacific Islander
Characteristics	*n* (%)	*n* (%)	*n* (%)	*n* (%)	*n* (%)	*n* (%)
Participants	8	8	10	8	8	4
Gender						
Male	0	4 (50)	5 (50)	5 (62.5)	2 (25)	1 (25)
Female	8 (100)	4 (50)	5 (50)	3 (37.5)	6 (75)	3 (75)
Country of origin	Iraq	Iraq	Iraq	Egypt	Cook Islands	New Zealand
	Lebanon	Syria	Syria	Iraq	New Zealand	Niue
				Lebanon	Samoa	Samoa
				Syria	Tonga	

### 2.5 Data Analysis

Workshops were transcribed verbatim by the BPHNs. The workshops for Arabic‐speaking participants were transcribed in Arabic and then translated to English by a qualified NAATI certified interpreter and translator. A synthesis of the themes was performed using NVivo software (QRS International) for coding and theme generation.

Thematic analysis was conducted using Consensual Qualitative Research [32] methodology and applying an asset‐based approach, with a focus on identifying both barriers to healthcare access along with the proposed solutions. The analysis was conducted with each transcript systematically reviewed and independently coded by two BPHNs (L.S. and L.K.) under the supervision of an experienced qualitative researcher (R.L.J.). Codes were initially generated through an open coding round, whereby segments of the transcript were labelled with descriptive codes representing themes. Two researchers then conducted cross‐checks to compare emerging codes at each step. Codes were then refined and grouped into higher‐order categories and then reapplied across each transcript in a closed coding round. Themes were agreed and finalised following review by a second experienced qualitative researcher (S.S.).

### 2.6 Reflexivity

The research team brought a range of professional and cultural perspectives to the study, which shaped the design, facilitation and interpretation of findings. L.S. and L.K. are BPHNs, born in Iraq and New Zealand, respectively, and with 1–2 years of experience supporting Arabic‐speaking and Pacific Islander/Māori communities. L.S. and L.K. are both women, and they co‐facilitated the workshops with C.S. and WS, who are both male, C.S. originating from New Zealand and W.S. from Syria. Years living in Australia for the BPHN team ranged from less than 1 to 10 years. Their trusted role enabled culturally safe engagement, though their positions as facilitators and researchers required careful attention to power dynamics (addressed through voluntary participation and anonymised data). S.S. is a clinical research nurse with over 25 years of experience. Her knowledge of healthcare delivery informed practical aspects of the study and ensured clinical relevance across diverse settings. R.L.J. is an Allied Health clinician with a Master of Public Health and a PhD in health literacy. With over 25 years' experience in hospital‐based care delivery and 10 years' experience in co‐design and qualitative research, she led methodological design and supported critical reflection on systemic barriers and equity. These varied perspectives enriched the research process, with regular team discussions used to reflect on potential bias and ensure rigour.

### 2.7 Data Reporting

We have reported our study according to the Consolidated Criteria for Reporting Qualitative Research (COREQ) [33].

## 3 Results

Analysis of the content arising from the workshops revealed five overarching themes—overcoming language barriers, improving communication, navigation and access to information, appointment reminders and health and social services education in the community. An overview of each of the barriers and proposed responsibilities, as well as which cultural groups identified this theme, is outlined in Table 2.

**Table 2:** Barriers and proposed solutions identified in the workshops.

Theme	Barrier Identified	Proposed BPHN tasks	Arabic‐speaking workshops	New Zealand Māori and Pacific Islander workshops
1. Overcoming language barriers	Language proficiency challenges prevent effective communication. Reliance on family or friends for interpretation, delays in accessing interpreter services.	Facilitate communication Provide language support Prepare patients for appointments Ensure access to translated materials	✓	✓
2. Improving communication	Use of medical jargon and unclear explanations leaves patients confused and excluded from decision‐making processes.	Simplify medical language Provide emotional support Enhance engagement Encourage consumer participation	✓	✓
3. Navigation and access to information	Patients lack knowledge about available services and struggle with physical navigation within facilities. Digital literacy and information overload also hinder access.	Guide physical navigation Share local health resources knowledge Assist with digital literacy Promote health literacy	✓	✓
4. Appointment reminders	Missed appointments and delays due to unclear or incomplete communication about scheduling, directions or follow‐up care.	Deliver culturally sensitive appointment reminders Provide visual aids Ensure patient follow‐up	✓	
5. Health and social services education in the community	Limited access to culturally relevant health education prevents effective health management and informed decision‐making.	Deliver educational workshops Leverage community spaces Create inclusive materials Encourage ongoing learning	✓	✓

### 3.1 Overcoming Language Barriers

Participants across all workshops identified that language barriers were one of the greatest challenges they experienced when accessing healthcare. Language difficulties hinder effective communication between patients and healthcare providers. Many community members rely on family or friends to interpret when facing barriers to accessing interpreter services. Even when interpreters are available, communication can still be inadequate, as patients struggle to comfortably express their needs in full. Participants across all workshops identified language as the most significant barrier to accessing healthcare. Box 2 provides an overview of the proposed tasks for a BPHN in assisting with overcoming language barriers to care. Participants described situations where they are dependent on family members or friends to interpret for them.

‘They have to have someone that speaks the language better and that means a child, the relative, a friend, and they can't have that all the times.’

Pacific Islander/Māori Workshop 1

Box 2 **Overcoming language barriers: Proposed responsibilities for bicultural peer health navigators.**

**Facilitate communication**

BPHNs can act as intermediaries to bridge language gaps, ensuring patients understand medical instructions and healthcare processes.

**Provide language support**

BPHNs can assist in coordinating interpreter services or provide language assistance if appropriately qualified.

**Prepare patients for appointments**

By coaching patients on key questions to ask and helping them articulate their concerns, BPHNs can enhance the quality of medical consultations.

**Ensure access to translated materials**

BPHNs can distribute and explain translated health information to improve understanding of medical instructions and appointment details.

Participants identified that even when interpreters were available, they still experienced challenges in clearly communicating their needs.

‘When you get a translator, it becomes difficult … to tell the translator what to say…. It's hard to express it to someone else.’

Arabic‐speaking Workshop 4

Participants also identified how the limited availability of interpreters and the lack of appropriately translated health information are barriers to accessing healthcare.

‘Providing a translator as soon as possible, not waiting for an hour or two until the translator comes to me.’

Arabic‐speaking Workshop 3

A BPHN could facilitate access to information in language, both in the written form and access to interpreters, to support the patient's understanding of medical instructions and ability to communicate effectively with healthcare providers.

‘Maybe they need something and don't understand the nurse or doctor; having help would be beneficial.’

Arabic‐speaking Workshop 3

### 3.2 Improving Communication

Participants used examples and stories from their own experiences to highlight challenges they experienced in trying to understand information communicated by the healthcare service. Healthcare providers often use medical jargon or fail to explain processes, leaving patients feeling confused and excluded from decision‐making. Participants also reported feeling isolated and uninformed while navigating the healthcare system. These experiences provide insights into the challenges faced by members of their communities with emergency department waiting times, hospital admissions and their understanding of treatments and care plans. Box 3 provides an overview of the proposed activities for a BPHN in assisting with improving communication.

‘And they don't tell you what they're doing, what's happening in the surgery. I mean, no one informed me about what they were doing; we just signed papers.’

Arabic‐speaking Workshop 3

Box 3 **Improving communication: Proposed responsibilities for bicultural peer health navigators.**

**Simplify medical language**

BPHNs can explain complex medical terms in plain language to help patients understand their diagnoses, tests, treatments and care plans.

**Provide emotional support**

By attending appointments with patients, BPHNs can act as advocates, clarifying medical advice and ensuring patients feel heard.

**Enhance engagement**

BPHNs can explain health service processes, such as triage systems in emergency departments, to better manage expectations and reduce frustration.

**Encourage consumer participation**

BPHNs can support patients in formulating questions to ask their healthcare provider.

Participants highlighted the importance of healthcare providers avoiding medical jargon and using simple terms.

‘[We need a doctor who] knows knowledge but can say it in a basic way.’

Pacific Islander/Māori Workshop 1

Participants also shared their frustration about their experience of isolation in the hospital setting, describing situations where they felt unattended, waiting with no updates or explanations about their situation or care progress.

‘You don't know the nurse from the doctor or who works in the hospital, you don't see anyone…. So, you don't know what's happening.’

Arabic‐speaking Workshop 3

A BPHN can act as a support person in appointments to enhance communication, help patients understand the access to healthcare processes, such as the triage system in the emergency department and how this might impact waiting times, as well as help them to understand their treatment and follow‐up plans better.

‘It's not just about the appointments; someone should also be there to explain what's happening.’

Arabic‐speaking Workshop 3

### 1 Navigation and Access to Information

Participants reported they often lack knowledge about available services and struggle with physical navigation within healthcare facilities. Additionally, digital literacy and information overload can prevent them from effectively accessing health information. Cultural diversity and language proficiency also influence an individual's ability to access and understand the healthcare system.

Participants identified that they are often unaware of services that may be available to them. Box 4 provides an overview of the proposed tasks for a BPHN in assisting with navigation and access to information.

‘I haven't been here that long, so I'm not sure what resources are out there or what support is out there for our Pasifika people.’

Pacific Islander/Māori Workshop 2

Box 4 **Navigation and access to information: Proposed responsibilities for bicultural peer health navigator.**

**Guide physical navigation**

BPHNs can meet patients at hospital entry points and accompany them to their appointments, reducing confusion and stress.

**Share local health resources knowledge**

Using culturally appropriate channels such as social media, community gatherings and printed materials, BPHNs can inform patients about healthcare services.

**Assist with digital literacy**

BPHNs can help patients navigate health systems for appointment scheduling and accessing test results.

**Promote health literacy**

Through workshops and one‐on‐one sessions, BPHNs can provide education about available services and how to access these effectively.

Participants suggested that information could be provided in their languages on social media platforms, advertising at local councils or community centres, or using traditional media, including newspapers and radio stations. Participants stressed the importance of sharing the information in multiple languages across multiple platforms to ensure inclusivity. Participants also suggested BPHNs could present at community gatherings, such as community groups and churches, to share information effectively and spread information through word of mouth.

‘Some of the things I'm thinking of is, you know, we already have some good avenues … embedded in the community, i.e. churches, community groups, we have Samoan Advisory Council. I think those are traditional methods of communicating. I don't know that they're always the best so I'm thinking like how do I find out stuff? It's always on Facebook, it's on “insta.”’

Pacific Islander/Māori Workshop 1

The opportunity for BPHNs in providing information was highlighted by participants. A BPHN could present information in a simple and easy‐to‐understand way, especially for the elderly or those with limited education or health literacy.

‘Yeah, I understand it. That's because I like reading things, but people who don't have that sort of education…. Yeah, elderly people be harder to comprehend with the information given to them would be harder.’

Pacific Islander/Māori Workshop 2

Participants highlighted the need for someone from their cultural background to support them in navigating the health system.

‘Sometimes it takes that navigator in the middle to be that stepping stone.’

Pacific Islander/Māori Workshop 2

BPHNs might not necessarily be able to directly help with these barriers, but participants did express the impact of effective communication between BPHNs and individuals in assisting in the identification of and directions to available services.

‘When there's communication between me and the health guide, the guide will provide services that I won't refuse, meaning they'll be helpful.’

Pacific Islander/Māori Workshop 3

### 1 Appointment Reminders

Participants in the Arabic‐speaking workshops identified that missed appointments and delays in care often result from unclear or incomplete communication about scheduling, directions or follow‐up care. Participants highlighted that appointment reminders reduced confusion, thereby reducing missed appointments and delays in care. The importance of having appointments and other information provided in language was also stressed, and where possible, visual aids to enhance understanding of how to navigate to appointments were also suggested. Box 5 provides an overview of the proposed tasks for a BPHN in providing reminders for appointments.

‘I received the appointment and went there. The information on the paper said, “Inquiries here,” but it wasn't clear. They said, “Go right,” but where to the right? The hospital is large, not a corridor or two, almost three‐quarters of an hour we were searching, and we found the place we want.’

Arabic‐speaking Workshop 3

Box 5 **Appointment reminders: Proposed responsibilities for a bicultural peer health navigator.**

**Deliver culturally sensitive reminders**: BPHNs can send reminders via text messages or phone calls in a patient's preferred language.

**Provide visual aids:** Maps, diagrams or other visual tools can help patients understand where to go for their appointments.

**Ensure patient follow‐up:** BPHNs can contact patients' post‐appointment to confirm their understanding of next steps and provide support in addressing any lingering questions.

Conversations also included suggestions for improvements, such as the use of visual aids, links to maps, directions within the hospital in language, and any other additional details needed for individuals to safely navigate the hospital and find their appointment locations.

‘There is a possibility for creating a “patient” map showing where they need to go.’

Arabic‐speaking Workshop 1

Participants also expressed their frustration over delays in receiving notifications of follow‐up appointments and test results. They reported confusion and dissatisfaction with the lack of communication about appointment scheduling and the overall management of a patient's healthcare needs.

‘About three months ago, I had an appointment with a specialist doctor. He asked me to do tests at the same hospital. I did the tests, and he asked me to do some imaging. Yes, an ultrasound, outside the hospital. He said he would schedule another appointment for me after ten weeks. It's been about three months, and no one has contacted me.’

Arabic‐speaking Workshop 3

### 1 Health and Social Services Education in the Community

Limited access to culturally relevant health education prevents individuals from managing their healthcare needs effectively and making informed decisions about care. Access to health and social services education in the community was identified as a need across both participant cultural groups. Workshops and group education sessions were suggested to assist community members in gaining a better understanding of their health conditions and how to access necessary support to manage their health conditions for improved health outcomes and quality of life. Box 6 provides an overview of the proposed tasks for a BPHN in providing health and social services education in the community.

Box 6 **Health and social services education in the community: Proposed responsibilities for bicultural peer health navigator.**

**Deliver educational workshops:** Collaborating with community leaders, BPHNs can organise workshops on topics such as chronic disease management and health promotion.

**Leverage community spaces:** BPHNs can use trusted venues like churches, schools and sports clubs to disseminate health information.

**Create inclusive materials:** Educational content can be tailored to community needs using multiple languages and formats to ensure inclusivity.

**Encourage ongoing learning:** BPHNs can motivate patients to attend future sessions and share knowledge within their networks.

Participants indicated healthcare providers, together with BPHNs and recognised community leaders, could determine the issues of highest need and support the development and presentation of education in formats and community facilities supportive of cultural norms.

‘And just to add an experience, there was a like workshop healthy eating and they've been amazing. I go to all of them.’

Pacific Islander/Māori Workshop 1

The Pacific Islander and Māori participants in particular emphasised the importance of using church groups, schools and sports clubs, citing that this is where members of their communities are likely to gather.

‘I think they really need to go into like, church groups, sports groups where you find a lot of our Polynesian are based on church groups, community groups, sports groups. This is where you're going to find all our Pacific Island. People are going to be associated with all these types of different community groups. And if we can put things in those areas, I reckon that'll be a good.’

Pacific Islanders/Māori Workshop 2

## 4 Discussion

This study explored factors impacting Pacific Islander, Māori and Arabic‐speaking communities in accessing healthcare in Australia. We identified five main themes across all the workshops: overcoming language barriers, improving communication, navigation and access to information, appointment reminders and access to health and social services education in the community. Based on these themes, responsibilities and tasks to develop the role of BPHNs were identified to address these barriers for members of these CALD communities.

While there was consistency in the themes raised across the workshops, the way in which they were experienced and prioritised varied across the cultural groups, highlighting the importance of cultural specificity in programme design. Arabic‐speaking participants frequently emphasised challenges with missed appointments, unclear follow‐up communication and limited access to appropriate interpreter services—reflecting structural and linguistic barriers that can compound over time and lead to disengagement. In contrast, Pacific Islander and Māori participants placed stronger emphasis on the need for culturally grounded engagement, with a particular focus on community‐based education and outreach through trusted spaces such as churches, schools and sports clubs. Despite higher levels of English proficiency in this group, participants also reported difficulty with complex health language and an underlying discomfort in articulating health needs within mainstream healthcare settings. This is consistent with research that has shown that language proficiency alone does not eliminate communication barriers between healthcare providers and patients [34, 35, 36]. These differences highlight the importance of tailoring BPHN roles not only to shared barriers but to the distinct social and cultural contexts in which different CALD groups experience the health system. In addition, it highlights that while professional interpreters play an important role in communication, their primary focus is on accurate message transfer—not on fostering understanding, building trust or providing continuity—making their role clearly distinct [18, 37].

Beyond describing barriers, our findings also point to broader structural and systemic issues that underlie many of the challenges reported. Appointment reminders were a key concern for participants. For many CALD communities, routinely used appointment reminder systems may not be fit for purpose due to language and health literacy barriers [9, 23, 38]. Appointment reminders that are not provided in a patient's preferred language can hinder comprehension, while unfamiliarity with healthcare systems and low health literacy may affect a patient's ability to act on these reminders [11]. The BPHN can play a very important role in overcoming these barriers by offering person‐centred, tailored support, such as providing reminders in the patient's preferred language, making phone calls to facilitate understanding of healthcare provider requirements and offering clear instructions for navigating healthcare facilities. Additionally, BPHNs can accompany patients to appointments if necessary, thereby ensuring effective communication and follow‐up and reducing the risk of missed or delayed care. This person‐centred approach helps bridge gaps and improves access in healthcare delivery for CALD communities.

Participants' stories across the workshops reflected a lack of agency and visibility within the system, where they were often passive recipients of care with little opportunity to ask questions or participate in decision‐making. This is in agreement with previous literature, which highlights the disempowerment experienced by consumers during consultations, with those from CALD backgrounds more likely than Australian‐born to defer to authority figures due to cultural norms, limited language skills or perceived power differentials [39, 40]. Such dynamics can contribute to disengagement, poorer communication and increased risk of misdiagnosis or inappropriate treatment [41]. Furthermore, institutional practices and models of care are often not designed with cultural safety in mind, reinforcing passive roles for patients from marginalised communities [11]. The presence of a BPHN—someone culturally aligned and accessible—can begin to shift this dynamic by acting as a cultural broker and advocate, supporting patients to express their needs and participate in decisions about their care. In doing so, BPHNs play an essential role in enabling more equitable engagement and fostering shared understanding between patients and providers.

Understanding and navigating a healthcare system that differs from the one in a patient's country of origin may also impact their ability to access care. Consistent with international studies, our findings highlighted barriers associated with navigation, such as health system design, limited access to healthcare providers from CALD communities, physical access to services, resource accessibility, and cultural barriers and expectations [18, 35, 38]. The presence of a BPHN was recognised by participants of the workshops as important in both assisting CALD community members in navigating healthcare and building trust between healthcare providers and their communities—a finding consistent across workshops. According to Harrison et al. the inability to ask for or use the required service is an identified barrier that leads to anxiety and reduced confidence for CALD consumers in seeking medical attention [35]. BPHNs can not only aid consumers in navigating and accessing the healthcare system but will also be a representative of that community—someone who shares their cultural background and may understand their unique needs for support. This connection can reduce barriers to accessing healthcare services, which is supported by earlier studies that have highlighted the benefits of interactions with individuals who share the same language, cultural understanding and knowledge of the healthcare system [9, 19, 42].

There are a number of implications for our findings for healthcare practice, policy and workforce, as well as education and training. Employing BPHNs can bridge communication gaps, improve community engagement and provide culturally and linguistically appropriate support. Employing BPHNs who demonstrate empathy and can provide culturally competent support is important for building trust and improving engagement with those from CALD communities. Addressing transportation and financial barriers through a BPHN can help mitigate these access barriers. Community‐based health education programmes co‐designed with community members and supported by BPHNs can enhance health literacy and outcomes for CALD communities.

## 5 Strengths and Limitations of the Study

A strength of this study is that it has been led by a team of BPHNs. We also used a co‐design methodology that included diverse participant representation, with over nine countries of origin represented across Arabic‐speaking, Pacific Islander and Māori communities. We conducted the workshops in the participants' language (Arabic), which ensured culturally relevant and detailed discussion highlighting community insights, and we conducted a member‐checking exercise to determine whether our findings accurately reflected discussions in the workshops. The involvement of BPHNs fostered a safe environment, while the focus on two distinct CALD communities highlighted common barriers and actionable solutions. However, these findings may be limited in generalisability due to the focus on only two cultural communities living in a single geographic area of Melbourne.

## 6 Conclusion

Delivering effective healthcare to CALD communities is complex, requiring targeted approaches to overcome persistent barriers. This study identified key challenges faced by Pacific Islander, Māori and Arabic‐speaking communities in accessing healthcare in Australia, including language barriers, communication challenges, difficulty navigating the healthcare system, appointment management issues and limited access to health education. By co‐designing the role of BPHNs, we proposed culturally tailored responsibilities to address these barriers, including facilitating communication, simplifying health care processes and promoting health literacy. BPHNs can act as a conduit between healthcare services and CALD consumers, fostering trust and empowering individuals to navigate their healthcare journey more effectively. Our findings reinforce the importance of recognising the unique needs and experiences of different communities rather than adopting a one‐size‐fits‐all approach. Integrating BPHNs into healthcare systems is not only a practical response to these challenges but also a critical step towards achieving equitable healthcare for all. Future research should focus on evaluating the impact of BPHNs in diverse settings and scaling this model to support other underserved communities.

## Author Contributions

R.L.J. conceptualised and designed the study and acquired funding. L.S., S.D.W., F.H., L.K., W.S., C.S., M.M. and R.L.J. conducted the investigation. L.S., S.S., L.K. and R.L.J. conducted the formal analysis. S.S., M.M. and Y.H. validated the data. S.D.W. and F.H. provided project administration. L.S., S.S. and R.L.J. drafted the manuscript. S.D.W., F.H., L.K., W.S., C.S., M.M. and Y.H. reviewed and edited the manuscript. R.L.J. provided overall supervision.

## Ethics Statement

Research ethics and governance approval by the Northern Health Ethics Committee and Office of Research (HREC/87830/NH‐2022). The guidelines of the Declaration of Helsinki were followed in every stage of this study.

## Consent

All participants provided their informed consent to participate in this study. All participants provided their voluntary informed consent for publication during the informed consent procedure.

## Conflicts of Interest

The authors declare no conflicts of interest.

## Data Availability

The data that support the findings of this study are available from the corresponding author upon reasonable request.
